# The Ethics of Truth-Telling in Health-Care Settings

**DOI:** 10.21315/mjms2018.25.3.14

**Published:** 2018-06-28

**Authors:** Yusrita Zolkefli

**Affiliations:** PAPRSB Institute of Health Sciences, Universiti Brunei Darussalam, Jalan Tungku Link, Gadong BE1410, Brunei

**Keywords:** truth-telling, health professionals, ethics, deception

## Abstract

Can a lie be justified if it saves a human life or a community, or if another great evil is avoided? The article proposes that health professionals need not always tell the truth, depending on situation; but, this does not refute the significance of telling the truth. It also elucidates the value of telling the truth, and the challenges for telling the whole truth. Two prominent theories of ethics, Deontological and Consequentialism are deliberated, together with the integration of examples to illustrate main areas of interest.

## Introduction

The truth hurts, as most people say. Yet while honesty has always been understood as the best policy, it has also played a role in the temptation to lie. Health professionals are expected to always tell the truth to their patients simply because it is the right thing to do. Still, arguably, if they were to examine their work every day, there are demands in which the truth is not always a definite matter. This brings us to the question: Is there a special moral duty and obligation for health professionals to always tell their patients the truth, or are there situations where some degree of dishonesty may be justifiable to avoid more serious harm to a patient? If there are reasons for not telling the truth, what are they? When could incomplete disclosure be justified, and under what circumstances? In the past, where the value of not doing harm (non-maleficence) was so strong, lying to the patient was considered acceptable whereby the arguments maintained that health professionals’ primary moral obligation was to help and not cause harm to patients. Therefore, lying was generally accepted, and news that is perceived as causing stress was withheld to avoid for what is judged as the best interest of the patient. Today, many things have changed, and telling the truth has emerged among the most widely praised qualities of health professionals in contemporary biomedical ethics (1).

## The Value of Truth-Telling in Health Care

One of the most pre-disposed values to being truthful is associated with respect for the patient as a person who is able to make decision. This is because, to determine a course of action and governance of care for a patient, the patient requires nothing less than truthful information. The provision of truthful information to patients is one way to enable them to make correct decisions which benefit their overall health. Without knowledge of the truth, it would be uncertain whether patients can make informed decisions and would lead to failure of health professionals to respect them as autonomous individuals. Lying is held to be a breach of the autonomy of the person, and this contradicts concepts such as patient empowerment, shared decision-making and patient-centred care. This is essentially significant to a health professional obtaining informed consent, whereby the potential risks involved in the proposed treatment and intervention need to be disclosed truthfully. To consent to any health intervention, a person requires sufficient and truthful information to make an informed and conscious choice; arguably, patients cannot make effective decisions without truthful information.

The second value is on duty and trust, whereby Kant (2), one of the leading Western philosophers, believed that everyone has a strict duty to tell the truth even if it might be harmful. He believed that lying could never be an excuse, as it was always harmful to a particular person or to mankind in general. If harm results from telling the truth in a compassionate manner, then it is an ‘accident’, but if harm results from a lie, then the liar is responsible. Kant further supposes that telling the truth is always a duty, whether it relates to the other’s right to know or results in innocent people being severely harmed. In other words, from a deontological point of view, competent patients should be told the truth regardless of the consequences. It can also be argued that, given the value of trust in any health professional–patient relationship, then such trust must be properly facilitated and fostered throughout patient care. This may not be possible if patients discovered that they have been deceived by health professionals who are otherwise trusted to not tell lies. Furthermore, while lying may be justified at times, its main effect soon becomes evident, that despite liars believing their actions to be benign and in good faith, those deceived may feel upset and wary (3).

The third value is the physical and psychological benefits of telling the truth. One of which is the positive benefits on the patient; those who are well informed tend to collaborate with health professionals and seek to be treated. This is based on the belief that once the patient knows their diagnosis and prognosis, they can tolerate the treatment and the pain more positively. Meanwhile, in the absence of disclosure, harm may result from not seeking treatment. We must not forget that telling rather than withholding information will allow a patient to plan their care, seek other opinions and put personal and financial affairs in order (4). Therefore, it is very difficult to think of a situation where lying can ever be acceptable in the therapeutic relationship. Moreover, honesty also helps protect the patient from overtreatment, which is neither kind nor beneficial (5). In terms of psychological benefit, knowing one’s prognosis and diagnosis is far less debilitating than worrying about the unknown because patients who are not given the opportunity to reveal their own fears and worries may be left anxious and convinced that they have the most horrible fate ahead (6). As a result, this avoidance of communication about the reality of a patient’s situation may actually expose the patient to considerable psychological distress. Concealment, once started, and even with good intentions, would probably have to be continued (7). There is some evidence to support the notion that informing patients the full truth about a life-threatening disease does not result in a greater incidence of anxiety, despair, sadness, depression, insomnia or fear (8). In fact, informed patients engage in better communication with health professionals, resulting in greater trust in the care provided. Furthermore, it is alleged that not informing patients of the natural course of their illnesses deprives them of what is called a ‘good death’ (9). If, for example, patients were made more miserable by being given information about their condition and risk of alternative treatment, if it is what the patients wish to know, then health professionals are morally obliged to tell them the truth.

## The Challenge of Truth-Telling in Health Care

Respect for patient as a person to be told the truth may possibly contradict with a patient’s right not to know such truth. In some cases, patients prefer not to be told or have full information of their health conditions, of a serious diagnosis, but would rather wish a family member be informed (10). In other words, some are happy not to be given the unpleasant information and are happy to leave the decision making to the health professional or family. Autonomous individuals are free to use their autonomy as they see fit, even to delegate it when this seems right, or if they find themselves unwilling or unable to cope with the information (8). For example, an elderly patient who had just numerous blood tests, was exercising her right to making decision, by asking that the doctor discuss the results with her daughter. To force or exert the truthful information on someone who might not be ready to deal with the impact of the information can be seen as oblivious and possibly damage the relationship between the health professionals, the patient and the family. For example, deception to a depressed, tearful patient who is in need of comfort may actually promote greater respect for autonomy than the oblivious truth. Successful deception may possibly infringe the patient’s immediate autonomy but does not mean it fails to respect the patient’s overall autonomy (11). Therefore, would recognising this wish symbolise a violation or respect to the patient’s autonomy? In any health care codes of ethics, it is generally established that health professionals ought to respect their patients’ wishes and preferences. Yet, this respect of wishes is not just about the patient’s right to know, but extends to respect a patient’s right not to know. This suggests that while there is a compelling argument for safeguarding respect for individual autonomy in being truthful, there is robust evidence emerging that such notion is not always absolute, particularly when a patient does not wish to be told the truth or to possess truthful information about them.

In the earlier arguments, it is part of health professional’s duty to tell the truth. Given the duty of openness and honesty is increasingly recognised as critical in any health professional relationship with patients, there are some uncertainties to such duties. For example, in patients with conditions of cognitive deficit such as dementia, it remains debatable if they are entitled to be told the truth in the first place. Generally it is perceived as justifiable to withhold information from certain patients and not tell the truth to patients who appear incompetent in accepting the information, or who have cognitive defects (12). It can also be argued that telling the truth is only a prima facie obligation, in other words, when there is conflict with other obligations, one can override the other obligation (1). This is in particular relevant to the principles of beneficence and non-maleficence which are used to justify for not telling the truth to patients. Previous arguments perceive that telling lies may potentially lead to physical and psychological harm, but what if, by telling lies offer greater benefits to patient, than causing harm? Should we then promote beneficence instead whilst taking into account our primary duty not to harm patients? Consider the following case study (13). There was a car accident whereby a man was badly injured while the family have been killed. This badly injured man regains consciousness in a hospital bed and he is critically ill and fighting for his life after a road accident. If this man were to ask about his family as soon as he has regained consciousness, would lying to him be justified? It certainly be difficult to see how it makes moral sense to tell him the truth, that his wife and three other daughters have been killed, until his condition is no longer critical, and the news, at that point, is unlikely to risk his life, although it would be a different matter if he were about to die. Therefore, perhaps it is good to reflect that if illuminating the truth would cause harm and a lie is told with the clear intention of achieving good, then lying can sometimes be morally justified. This is also based on the consequentialism point of view, insisting that the decision to tell or not to tell the truth depends on the details of the clinical situation, and the doctor should decide which course of action might be least harmful in producing the best results for the patient (7). It is further argued that there is a difference between ‘telling the whole truth” and “giving a patient a true picture”. Since health professionals involve specialist knowledge, therefore to tell the client “the whole truth” about a particular condition, explaining the biochemistry, physiology, and histories of like conditions in other people, might not be pragmatic. It is also impossible to provide the patient with such knowledge particularly where there may be little time whilst proper understanding might require the patient to have considerable prior knowledge. To some extent, patients will vary in their ability to understand the complexity of medical information and of course, ‘the whole truth’ is usually an illusion (8). Giving the patient a ‘true picture’ about their condition or medical treatment is more pragmatic than telling them the whole truth, as inevitably, the most relevant points will be selected by the health professionals to tell the patients (13). Besides the truth can be ambiguous, situational and personal, and that telling the truth depends on how each of the health professionals define what ‘truth’ actually is.

Meanwhile, from a utilitarian’s perspective whereby the emphasis on the maximisation of the happiness and interests of all concerned, then perhaps not telling the truth is arguably justified in certain condition. For example, patients are not always necessarily told that novice doctors are performing treatments, nor are they informed of the risks associated with treatments performed by novice doctors. As greater benefits, via an increased physical knowledge from treatment, are achieved for a large number of future patients when a novice doctor practices a procedure. To disclose this would potentially discourage patient participation and reduce the learning of the profession, would impact on future practice and treatment outcomes (14). Another example is when a nurse, who puts medication to a patient’s food, is an elderly patient with cognitive impairment, being acutely disturbed and represents a significant risk of harm to them self or to others. Of course, the issue of covert administration of medication given to an autonomous individual against his or her will is both legally and ethically unacceptable (15). The presence or the true nature of the medication was denied because truthful disclosure would cause the patient to refuse the drug, which could result in a negative outcome with regards to the patient’s treatment. Therefore, could the benefit of giving, for example, a sedative outweigh the possible harm caused? Indeed, telling the patient the truth can sometimes appear to be more harmful to the patient and it may well be justified for the nurse to withhold information or even use benevolent deception (7).

## The Best Way Forward

Whilst most health professionals are always keen to treat patient with honesty, they must also carefully recognise and reflect on the assumption that all patients wish to receive truthful information directly, particularly when patient preferences do indeed vary. The meaning of truth and acceptance of it means differently across cultures, which necessitates treating people sensitively and sharing information using excellent communication skills. Health professionals also ought to approach cases on an individual basis and handle them delicately through careful deliberation and dialogue with the patient, their family and other multidisciplinary professionals. In cases where the overall welfare and long-term autonomy of the patient may possibly be maximised by means of deception, then such action must be clearly documented with justifications, and the decision must be reviewed on a regular basis. In the context of dementia care, for example, health professionals should seek to understand their patients’ preferences and act according to their choices rather than routinely disclosing or concealing such information. Patients with dementia require affirmation because they are individual people who may neither fully understand nor remember the truth, which could be challenging for health professionals (16).

At the same time, health professionals must be cautious of giving too much (truthful) information, as it can be overwhelming for some, if not all. It is common for people to misinterpret or misjudge new or too much information given to them, and therefore, health professionals must take note of considerations to recognise factors that can affect patients’ responses to information given. All this is necessary so that health professionals are encouraged to avoid undermining the obligation to be honest and truthful and to ensure that robust strategies are in place to effectively deliver information. At the same time, as part of addressing the psychological needs of the patients, preserving hope is seen as essential for patients to carry on with their life as normally as possible, and this may occasionally be maintained through avoiding certain information.

The above case examples direct medical doctors to carefully consider and bridge the cultural context and dimension as a salient point. Not only is there a need for established good rapport with patients, but increased awareness and understanding of cultural differences in truth-telling also helps frame the ethics of truth-telling. Such cultural sensitivity allow doctors to respect and accept the patient’s values, religious and cultural beliefs, whilst at the same time echoed on the significance availability and benefits of appropriate psychosocial, spiritual and religious support mechanisms (for example, clinical psychologist or counsellor). This could be one of the positive approaches to provide great support in doctor-patient cross-cultural communication and decision-making, hence giving patients the best care possible.

## Conclusion

Health professionals are expected to always tell the truth. This is based on the argument that, lying is wrong and disrespecting the person’s autonomy is not right. However, this may not necessarily be the case, as the ‘right not to know’ the truth, should as well be respected by them. In the discussion, it appears that the truth is an essential moral good, but, sometimes truth does come into conflict with other essential moral good like beneficence, nonmaleficence and autonomy. When conflict arises, a line ought to be drawn between respecting one’s autonomy for the truth of information and the promotion of the principles of beneficence and non-maleficence. Whilst physical and psychological implications of telling the truth to patients are addressed accordingly, it needs further consideration on both the harm of lies and the harm of telling the truth. Each patient nevertheless must be approached individually, and at a level that addressed his or her needs and interests. Hence, having considered the arguments where telling the truth stands in health care practice, health professionals may need not always tell the truth. These however necessitate them to not disregard the importance of telling the truth to patients and that in all situations, justification is needed before applying the notion of telling the truth.
